# The use of digitally collected patient-reported outcome measures for newly operated patients with total knee and hip replacements to improve post-treatment recovery: study protocol for a randomized controlled trial

**DOI:** 10.1186/s13063-020-04252-y

**Published:** 2020-04-09

**Authors:** David Kuklinski, Laura Oschmann, Christoph Pross, Reinhard Busse, Alexander Geissler

**Affiliations:** Technical University Berlin, Straße des 17. Juni 135, 10623 Berlin, Germany

**Keywords:** Health-care policy, PROM, Quality of care, TKR, THR, Value-based health care, Randomized controlled trial

## Abstract

**Background:**

The number of total knee replacements (TKRs) and total hip replacements (THRs) has been increasing noticeably in high-income countries, such as Germany. In particular, the number of revisions is expected to rise because of higher life expectancy and procedures performed on younger patients, impacting the budgets of health-care systems. Quality transparency is the basis of holistic patient pathway optimization. Nevertheless, a nation-wide cross-sectoral assessment of quality from a patient perspective does not yet exist. Several studies have shown that the use of patient-reported outcome measures (PROMs) is effective for measuring quality and monitoring post-treatment recovery. For the first time in Germany, we test whether early detection of critical recovery paths using PROMs after TKR/THR improves the quality of care in a cost-effective way and can be recommended for implementation into standard care.

**Methods/design:**

The study is a two-arm multi-center patient-level randomized controlled trial. Patients from nine hospitals are included in the study. Patient-centered questionnaires are employed to regularly measure digitized PROMs of TKR/THR patients from the time of hospital admission until 12 months post-discharge. An expert consortium has defined PROM alert thresholds at 1, 3, and 6 months to signal critical recovery paths after TKR/THR. An algorithm alerts study assistants if patients are not recovering in line with expected recovery paths. The study assistants contact patients and their physicians to investigate and, if needed, adjust the post-treatment protocol. When sickness funds’ claims data are added, the cost-effectiveness of the intervention can be analyzed.

**Discussion:**

The study is expected to deliver an important contribution to test PROMs as an intervention tool and examine the determinants of high-quality endoprosthetic care. Depending on a positive and cost-effective impact, the goal is to transfer the study design into standard care. During the trial design phase, several insights have been discovered, and there were opportunities for efficient digital monitoring limited by existing legacy care models. Digitalization in hospital processes and the implementation of digital tools still represent challenges for hospital personnel and patients. Furthermore, data privacy regulations and the separation between the in- and outpatient sector are roadblocks to effectively monitor and assess quality along the full patient pathway.

**Trial registration:**

German Clinical Trials Register: DRKS00019916. Registered November 26, 2019 – retrospectively registered.

## Background

Total knee replacements (TKRs) and total hip replacements (THRs) are among the most frequent and increasing surgeries in high-income countries, such as Germany. They are considered to be effective but also highly invasive procedures to treat osteoarthritis in the knee and hip. In 2016, in Germany, there were more than 187,000 procedures (230 per 100,000 population) for TKRs – a 38% increase compared with 2006 – and 233,000 procedures (280 per 100,000 population) for THRs – a 17% increase compared with 2006 [1–3]. Moreover, the number of primary as well as revision procedures is expected to rise steadily because of increased life expectancies and procedures performed on younger patients [2]. This development has a significant impact on the budgets of health-care systems. Measuring patient-reported quality helps to generate transparency, to evaluate treatments, and as a consequence to optimize patients’ pathways (indication, procedure, and recovery), leading to enlarged revision horizons and a decreased number of revisions [4].

Quality of joint replacement in Germany is measured and monitored by several mandatory or voluntary initiatives. They concentrate on the collection and analyses of clinical quality indicators and administrative data as well as the adherence to structural and processual standards and the interpretation of medical documentation [5]. These quality initiatives are limited mostly to the acute inpatient sector [6]. A nation-wide standardized and cross-sectoral assessment of quality from a patient perspective does not exist yet. As a result, the improvement of arthroplasty-relevant problems such as post-operative joint functionality, pain, or constraints in daily life cannot be assessed by the existing quality measurement tools. However, an improvement in those quality dimensions defines treatment success [7] and significantly affects patient utility [8]. Consequently, a recent study in the UK concluded that, for patients with THR, quality measured by patient-reported outcomes (PROMs) significantly affects patient utility in the form of willingness to travel and hospital demand positively. In contrast, existing quality indicators such as mortality rates or readmission rates have either only a small positive or even insignificant effect [8].

Furthermore, increased transparency of the patients’ recovery process using PROMs initiates a direct feedback mechanism between physician and patient, thus optimizing surgeries [9, 10] and best practice sharing [7]. At present this is barely given. After rehabilitation, patients will seldom return to the hospital for follow-up visits and instead they see their family physicians or outpatient specialists. Unfortunately, communication channels between hospitals and follow-up physicians are often not present, preventing any feedback mechanism regarding their patients’ recovery process [11].

For example, the effects of improved medical quality by continuously monitoring patients after surgery using PROMs have been concluded in a study of patients with cancer [12, 13]. The study shows that, during routine treatment, constant monitoring and early detection of critical recovery paths via PROMs significantly improved survival rates and decreased the number of emergency visits [12, 13].

Our study is designed to go beyond testing the effectiveness of PROMs as a quality measurement instrument. For the first time in Germany, we want to test whether an early detection of critical recovery paths after TKR/THR via PROMs and change of post-treatment pathways improves clinical quality indicators such as readmissions and patient-centered quality measures such as health-related quality of life and joint functionality and, in turn, leads to a decrease of health-care costs. The study focuses on two specific questions: (a) Can the use of PROMs after TKR/THR impact the patient pathway in a positive and cost-effective manner? (b) Can we recommend the implementation of PROMs into standard care, and what are best practices to follow?

## Methods/Design

### Overall study design

This study is a two-arm multi-center patient-level randomized controlled trial (RCT). Patient-centered questionnaires are employed digitally to regularly measure PROMs of patients with TKR and THR from the time of hospital admission until 12 months after discharge. With the support of an expert consortium of physicians, PROM threshold alert values at 1, 3, and 6 months have been defined to signal critical recovery of patients after TKR/THR. With the help of an algorithm, patients and post-treatment physicians are alerted to intervene in case of critical PROM values.

By adding cross-sectoral insurance claims data (resource consumption: drugs, physiotherapy, specialist visits, complications, infections, and hospital stays) at the patient level, the study examines the effect of PROM-based monitoring of patients up to 12 months post-surgery on the clinical as well as patient-reported outcomes and its cost-effectiveness.

### Ethics, consent, permissions, and funding

The study is funded by the Innovation Fund of the German Federal Joint Committee (G-BA) in the stream “Care models with comprehensive and measurable results and process responsibility”. The funding period is set between April 1, 2019 and March 31, 2023. The study will be conducted in accordance with the Declaration of Helsinki. The study was approved primarily by the Charité’s Ethic Committee, Berlin (EA4/169/19). The other responsible ethical review committees of participating hospitals (Medical Chamber Hamburg, Medical Chamber Schleswig-Holstein, Hannover Medical School, Friedrich-Schiller University Jena, and Medical Chamber Brandenburg) agreed with the decision. The study is registered at the German Clinical Trials Register (DRKS) under trial number DRKS00019916. All potentially eligible participants will be approached to offer their informed consent to participate in the study. The current protocol is version 1, dated January 15, 2020. Any changes in the study design will be communicated to all project partners, including hospitals.

### Study setting

The study started on October 1, 2019 and will last for two years, whereas patient recruitment will end after one year, on September 30, 2020. The second year will concentrate on follow-ups and corresponding interventions. After two years, the study will be evaluated by an independent and impartial research institute (aQua Institute, Göttingen, Germany). Study centers are the nine participating hospitals. These hospitals are located around Germany (seven different federal states) and are among Germany’s leading endoprosthetic centers. Hospitals differ in their type, including university hospitals, specialized endoprosthetic centers, full-service providers, and specialist outpatient clinics.

Based on historical hospital case volumes for elective TKR/THR to estimate expected workload, up to two study assistants are employed at each center – funded by means of the Innovation Fund – to operationalize the study design in each center. Before the start of the project, these study assistants were trained thoroughly in workshops to be in line with the specifications of the study design. The study assistants are responsible for all study-related activities during the patients’ hospital stay and for follow-up as well as the initiation and documentation of interventions and a complete final documentation of the participant after 12 months.

The study assistants and patients use a software interface provided by HRTBT Medical Solutions GmbH (Berlin, Germany), a digital solutions provider for hospitals, to document patient enrollment, key procedure aspects, and the patient-reported outcomes. Patients’ health procedure and claims data along the care pathway are accessed via the participating German sickness funds BARMER and BKK, both of which are part of the social health insurance scheme [14]. These insurance claims data will be extracted for four years (2018–2021) for those patients insured in one of these funds and having consented to study design and data sharing.

### Sample size

The estimation of the study’s sample size is based on historical case volumes (identified by procedure codes OPS 5–820 and 5–822) of the nine study hospitals from 2018. Hence, we expect around 17,500 patients to be eligible in the 12-month recruiting time frame. This figure already excludes emergency patients and minors. Furthermore, prior field studies by HRTBT Medical Solutions GmbH suggest that around 15% of patients reject participation and around 30% of the remainder will not finalize all follow-up surveys despite reminders and active follow-up efforts. Consequently, we expect to reach a sample size of around 10,500 participants having completed all follow-ups. This number is not yet adjusted for people without direct or indirect access to the internet or other technical constraints, but increasing case volumes compared with 2018 are expected to balance this effect.

Furthermore, it is expected that insurance claims data for patients insured in the two participating sickness funds (about 2700 patients) can be obtained, resulting in a sample size of 2700 for our primary endpoint. If we assume a statistical difference in effect between the intervention and control groups of 0.15 standard deviations, an error probability of less than 5%, and a power of 80%, a sample size of only around 1400 patients with insurance claims data is required for analysis.

### Eligibility criteria

Eligible for this study are all patients with primary elective TKR and THR above the age of 18 having a scheduled surgery or pre-surgery visit between October 2019 and September 2020. Exclusion criteria for participation are emergencies (e.g., femoral fracture), patients with American Society of Anesthesiologists classification 4–6 (4: a patient with a life-threatening disease, 5: a moribund patient who is unlikely to survive without surgery, 6: a deceased patient with confirmed brain death or an organ donor), and patients younger than 18 years. Additionally, for practical reasons, patients without direct or indirect access to an e-mail account are excluded from the study. E-mail addresses from relatives are accepted. All patients who deny participation will not be included in the study.

### Informed consent

All patients with a primary elective TKR or THR will be approached by their corresponding study assistants for a patient briefing once they have been scheduled for surgery in the corresponding hospital. Patients will be informed by either the study assistant or the corresponding physicians of the study and its benefits, risks, and design. Additionally, they are handed a comprehensive study information document. Before patients are included, they are required to sign an informed consent form for their study participation (Supplement 1). The informed consent allows us to contact the patient via e-mail, post, and telephone and to process and link the patient’s data from different sources during and after the study.

Patients are asked to sign two additional consent forms: (a) one for processing their hospital administrative data and (b) one for processing their insurance data in case the patient is contracted with one of the participating sickness funds. Both of these consent forms are optional and are not required to participate in the study.

Patients will be free to withdraw from either one of the three consents without stating a reason. If the patients withdraw from the consent for participation, all their data will be deleted. Patients are allowed to participate in other studies as long as these do not interfere with our study.

### Participant timeline

**Fig. 1:**
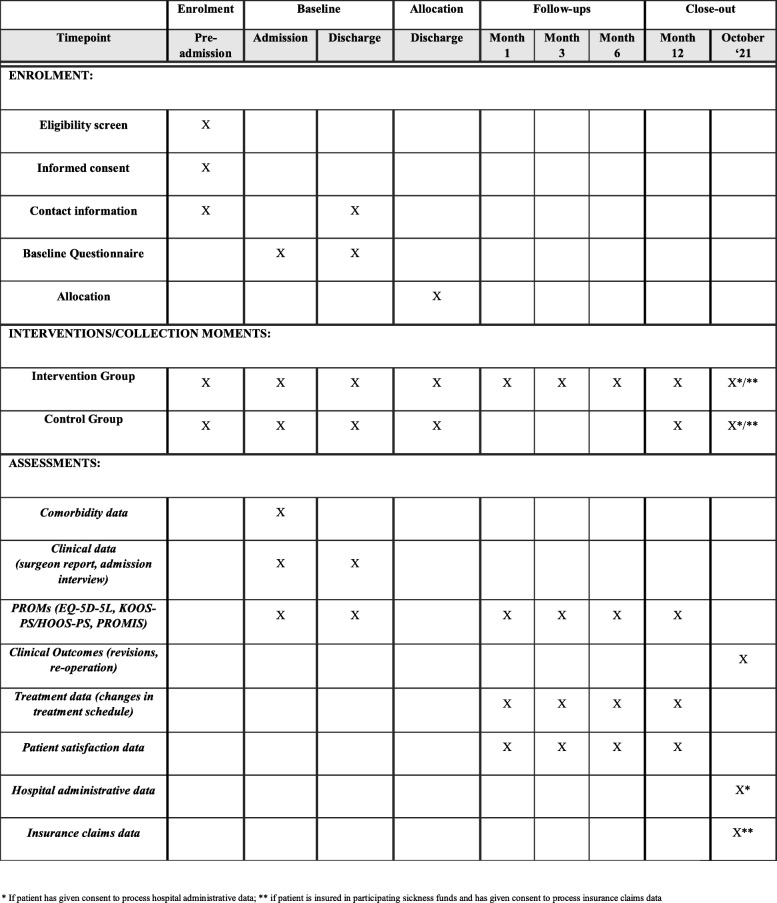
Standard protocol items – PROMoting Quality

Each participant will be participating in the study for a maximum of 12 months plus the duration of the hospital stay, starting with the admission day followed by the inpatient stay and the discharge and post-discharge period. The participant will have to answer two questionnaires during the hospital stay: at admission and discharge. At discharge, the randomized allocation into the intervention and control groups takes effect, and the participant is followed up with electronic questionnaires either one or four times in the 12 months after hospital discharge. With the final questionnaire after 12 months, the study will conclude for each participant regardless of allocation. The timeline for the participants is presented in Fig. 1.

### Baseline assessment

Baseline assessment takes place for all eligible participants at the day of admission into the hospital. The timing differs slightly between the study centers. Three study centers perform the baseline assessment during their pre-surgery examination days; this can be up to 6 weeks before surgery. After giving informed consent, the participant will be included in the study by providing name and e-mail address. In the next step, while waiting for the physician’s appointment, the participant is given a tablet to respond to a 10- to 15-min questionnaire consisting of basic demographic, comorbidity information, and selected PROM sets.

The overall questionnaire is based on the ICHOM (International Consortium for Health Outcomes Measurement) Standard Set for Hip and Knee Osteoarthritis, which consists of the EQ-5D-5L, HOOS-PS/KOOS-PS, and an analogue pain scale. This set was refined by adding the PROMIS Depression and Fatigue sets, as studies show that the recovery process is also affected by patients’ mental conditions [15]. During selection of the PROM sets, three criteria were of utmost importance. First, the questionnaire should be brief to include it in the hospital’s daily activities. Second, the sets should be renowned and proven by literature. Finally, the combined set of PROMs should cover all important patient-related aspects of the osteoarthritic condition and the elective TKR and THR procedures: health-related quality of life, functionality, pain, and mental condition.

At discharge from the hospital, the participant once again is handed a tablet to complete – alone or with the help of the study assistant – a mostly identical questionnaire (omitting demographic and comorbidity questions). Besides documenting the data from the questionnaire, the study assistant will document additional parameters at admission, after surgery, and at discharge, encapsulating information on the participant’s medical history, the surgery, and aftercare. In Fig. 1, the standard protocol items at different points in time are shown.

### Randomization and blinding

**Fig. 2:**
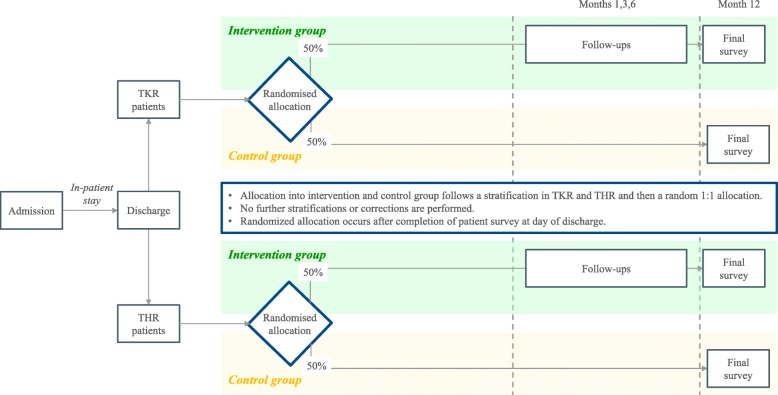
Randomization process

The allocation of participants occurs after completion of the patient survey at the day of discharge; thus, during inpatient stay, all eligible patients are treated equally. All participants are stratified into patients with TKR or THR and then randomly allocated at a ratio of 1:1 into the intervention or control group. No further stratifications or corrections are performed. Owing to data security regulations, randomization will be carried out for each hospital individually. Figure 2 shows the randomization flow. The study’s software provider will be responsible for the randomization algorithm, and allocation will be shown to the study assistant or the physician only after discharge of the participant. Hence, during the inpatient stay, the hospital staff and the participants are blinded. The participants will stay blinded until the end of the study, not knowing whether they are in the intervention or control group. Frequency of the surveys is not disclosed to patients, in either the intervention or control group.

### Intervention

Whereas participants in the control group will be asked to complete only a final follow-up questionnaire 12 months post-discharge, participants in the intervention group have to complete four follow-up questionnaires: at months 1, 3, and 6 and a final follow-up 12 months after discharge. All follow-up questionnaires are sent via e-mail and are answered electronically by the participants on their computers, smartphones, or tablets. In case the participants do not have an e-mail account, e-mails are sent to relatives if agreed upon and jointly filled out by relative and patient. Results from the questionnaires will be automatically transferred to the study’s software and are observable by the hospitals’ study assistants and physicians; 1-, 3-, and 6-month PROM set scores will then be screened for critical values or unfavorable developments.

When critical values or developments are detected, the software alerts the study assistants of the corresponding hospitals and an intervention is initiated. Interventions are characterized by three steps. First, the study assistant sends a standardized report to the participant, via post or e-mail, where critical values and developments are highlighted and a follow-up call is announced. Second, the study assistant calls the participants by phone to inform them of the critical values, asking permission to forward the PROM results to their outpatient specialist or general practitioner and recommending a visit to their physician to potentially adjust the treatment. Finally, a report with the participant’s results will be forwarded, by post or fax, to the outpatient physician. In the next regular questionnaire, participants are asked to report changes in their treatment schedule in order to document modifications in the outpatient physicians’ treatment with respect to physiotherapy, medication, or surgery.

For health-related quality of life (EQ-5D-5L) and functionality (KOOS-PS and HOOS-PS), critical absolute values and unfavorable development in the recovery paths were defined by an expert consortium of 13 orthopedic physicians, representing all nine participating hospitals. For this definition, the standard Delphi technique was deployed. The advantage of the standard Delphi technique is that it offers a structured process of collecting expert opinions, approaching consensus, without being biased by the domination effect of a few experts in open discussions [16]. The physicians separately populated templates for each item of the PROM sets – EQ-5D-5L (for hip and knee) and KOOS-PS/HOOS-PS at 1, 3, 6, and 12 months after surgery – subject to answering the following question: “Which patient value of this particular item would be considered critical at 1, 3, 6, or 12 months after surgery to schedule an appointment for a follow-up examination?”

Results were collected in one-on-one interviews, consolidated, and analyzed, and basic statistics were played back to the physicians, offering them the chance to adjust their values. Feedback was collected and once again consolidated, and results were played back and discussed in a final joint call. Moreover, it was agreed that neither the EQ-5D-5L nor KOOS-PS/HOOS-PS scores should worsen over time. Defined values have additionally been sanity-checked on historic values of patients of two participating study centers in order to test their sensitivity. For the PROMIS Fatigue and Depression scores, only absolute critical values were set on the basis of existing research; for example, a critical PROMIS Depression score was set to the PHQ-9 (Patient Health Questionnaire-9) classification of “moderately severe” depression [17]. All critical values are summarized in Table 1.

**Table 1:** Summary of critical values

PROM Sets	Month 1	Month 3	Month 6	Critical developments*
EQ-5D-5L hip	0.37	0.64	0.74	Yes
HOOS-PS	53.00	36.30	27.70	Yes
EQ-5D-5L knee	0.36	0.51	0.70	Yes
KOOS-PS	51.60	43.60	33.60	Yes
PROMIS Depression	65.80	65.80	65.80	No
PROMIS Fatigue	69.00	69.00	69.00	No

*Abbreviations*: *EQ-5D-5L* EuroQoL 5-dimension 5-level, *HOOS-PS* Hip disability and Osteoarthritis Outcome Score-Physical Function Short-form, *KOOS-PS* Knee injury and Osteoarthritis Outcome Score-Physical Function Short-form, *PROM* Patient-reported outcome measure, *PROMIS* Patient‐Reported Outcomes Measurement Information System

* Alerts at critical development defined as worsening score compared with previous questionnaire

### Data quality enhancement

Each study assistant is provided with a digital dashboard showing open tasks, critical value alerts, and patients’ activity related to receiving and answering follow-up questionnaires. Each task is associated with a deadline, and a color code reminds the study assistant of open tasks. For the purpose of data completeness, the participants will be reminded by the software to fill in their follow-up questionnaires 1, 2, and 7 days after the initial follow-up questionnaires are sent out. If a participant has not completed the questionnaire after three reminders, the study assistant will call to support him or her.

Moreover, predefined standardized e-mails, guidelines for the follow-ups, and intervention calls ensure consistency between study centers. Furthermore, adherence to the protocol given in the study design was promoted in training on the ground and during a central workshop for all study assistants and physicians.

### Hospital administrative data, clinical quality data, and insurance claims data

After completion of the last participant’s questionnaire and only for those who have given consent, hospital administrative data and insurance claims data will be added to the participants’ PROM data. Hospital administrative data are provided by the hospitals’ controlling department on the basis of the hospital internal case number of the patient. Insurance claims data are provided for participants who are insured at one of the participating sickness funds and have given consent.

### Outcomes

The overall aim of the study is to test the cost-effectiveness of PROMs for early identification and therefore timely treatment of post-surgical complications or the health-related quality decreasing developments or both. The primary endpoint of the study is to investigate cost-effectiveness and thus the optimization of outcome quality compared with the resource utilization of the designed intervention across the pathway. Outcome quality is defined as a composite measure of PROMs and clinical outcome measures such as re-operations. Utilized resources are direct and indirect follow-up health-care cost and the cost of implementing the designed intervention.

Secondary outcomes include the improvement of the patients’ functionality reflected by the KOOS-PS/HOOS-PS; of medium- to long-term health-related quality of life evaluated by the EQ-5D-5L; of pain in knee, hip, and lower back; of satisfaction with treatment outcome (measured through selected questions from the Patient Experience Questionnaire, or PEQ); and of clinical outcome measures such as post-operative revision and re-operation rates. Furthermore, these analyses will be repeated with subgroups. Specific analyses will be carried out to estimate the impact of enhanced recovery programs or the choice of a specific implant on patient-reported outcome quality, for example.

### Statistical analysis

Data will be provided in electronic formats. A statistical analysis program will be used to run tests and analyses. For the planned statistical analyses, it has to be distinguished between the primary and the secondary outcome.

For primary outcome – the cost-effectiveness of using PROMs in TKR/THR – a cost-effectiveness analysis (CEA) will be conducted. In the CEA, alternatives will be evaluated by costs and consequences (quality improvement) and mapped on a cost-effectiveness plane [18] and thus create support for later policy recommendations. For costs, the cross-sectoral sickness funds’ claims data on the patient level for direct and indirect follow-up costs as well as the cost of the intervention are taken into account to calculate incremental cost. For the patients’ overall health status, a composite measure is used. Results for each patient’s quality indicator are gathered, standardized, and combined into a quality index. This composite measure is a formative construct as it is expected that the different quality indicators will correlate [19]. In order to test for the robustness of the results, scenario or probabilistic sensitivity analyses or both are planned.

For the secondary endpoints, we will apply mostly multiple regressions using the differences in the quality indicator outcomes and improvements between the intervention and control groups. Several subgroup-level analyses for distinct and relevant patient cohorts will be performed. Besides others, subgroups will cover age, sex, education, mobilization, comorbidity, and type of prosthesis.

### Data management

The collection, storage, and processing of personal data in this study are carried out in accordance with the applicable data protection regulations of the federal states in connection with the European General Data Protection Regulation (GDPR) and take into account the specific provisions of the German Social Code, Tenth Book (SGB X). During the study, all data recorded electronically or in the hospital will be stored at the respective hospital servers and can be accessed or decrypted only by authorized users in the hospital environment. Data will be transferred to a secured personalized access SFTP-Server of the evaluating institute. Every authorized user is assigned to a separate area on the server where transport-encrypted (AES-256) data can be stored and retrieved.

All transferred data will be completely pseudonymized. Consequently, to match primary patient data to hospital administrative data and insurance claims data, a pseudonym will be created by the study’s software following UUID (universally unique identifier) standard and is added to the patient data. The pseudonym will be added to the hospital administrative data and insurance claims data. All other identification data will be deleted before it is transferred to the researching institute. Pseudonymized data will be stored for two years at the researching institute (Technical University Berlin, or TU Berlin) and stored at study centers for 10 years after the study completion to ensure further evaluation of study results. This complies with the recommendation for good practice for secondary data analysis [20].

### Study management

Study management is led by the project team at TU Berlin. The overall project team consists of the person responsible for the study design, members from the two participating German sickness funds, the technical provider, and all study assistants. The team meets on a regular one-month basis by teleconference to discuss the progress and solve arising problems. Regular one-on-one calls are conducted between the TU Berlin project team and study assistants to ensure that enrollment is in line with the study protocol. Additionally, on-site visits are planned every 3–4 months. During these sessions, study management will monitor adherence to the study design.

Furthermore, intermediate reports with statistics on recruitment and follow-up rates are sent to the study management each month. Regular status updates are sent to the study sponsor. Intermediate reports contain only aggregated data without participant-specific data. After 6 and 12 months, patient data sets will be extracted from the software to check data quality and consistency. Moreover, payouts to the hospitals are linked to patient recruitment rates (based on their internally projected yearly case volume) and follow-up completion rates. Therefore, study assistants need to export statistics at given points in time to receive full compensation. Owing to the character of the study, no adverse effects are expected, but unfavorable developments will initiate an intervention and be part of the study design.

## Discussion

Recent studies have shown the value of PROMs in monitoring patients’ recovery paths in oncologic settings [12, 13] as well as their effectiveness in measuring patient-relevant quality after TKR/THR [7, 8]. However, to the best of our knowledge, no RCT has examined outcome improvement and cost-effectiveness of monitoring and intervening in case of critical recovery paths with the help of PROMs.

### Insights

Depending on a positive and cost-effective impact of the intervention, a further aim is to evaluate the feasibility of implementing regular and digitized PROM-based communication into standard care. Furthermore, results from this study can be leveraged to define and implement selective contracts between sickness funds and health providers based  on quality indicators [21]. The setup phase of our study revealed several insights.

First, digitized PROMs can be a benefit but also a challenge to hospitals as well as patients. Unfortunately, the collection of PROMs on paper has been not effective and there have been low follow-up return rates and the burden of additional documentation work for hospitals. Digitized PROMs promise higher return rates and significantly reduced documentation work. Nevertheless, hospital IT departments need to be collaborative and personnel need to be trained to familiarize patients with digital tools.

Second, structural changes are needed to enable seamless implementation of the study design. The effectiveness of PROMs requires acceptance by physicians as well as hospital management. It could be observed that a move toward patient centeredness was generally perceived as very positive by physicians and study assistants. Nevertheless, resources are required for personnel who familiarize patients with PROMs and their relevance in the aftercare and who compile the data. This relationship between study assistant and patient is the central lever for data quality and completeness. Moreover, effective communication channels between hospitals and outpatient physicians and patients are key for the best possible recovery pathway. Often, this communication is disrupted because of the boundaries between the siloed in- and outpatient sector in the German health-care system.

Third, data privacy and security regulation were challenging factors in the setup of the study. New data security regulations complicate the communication between hospitals, patients, and outpatient physicians. A common nation-wide health data platform for collecting data and for communicating results could be an interesting idea for further discussion.

Lastly, in Germany, there is a diverse landscape of hospitals, ranging from full-service providers to specialized hospitals and specialist outpatient clinics. The diversity of the set of hospitals in the study sample already indicates that a standardized “one size fits all” process will not work. The collected insights in hindsight can be leveraged for further analysis to define the degree of standardization in the approach.

### Limitations

There are a number of limitations to the study at hand. First, owing to data security regulations and complexity, outpatient physicians are not formally part of the study. Unfortunately, we can only advise but not oblige the outpatient physicians to use the participants’ critical PROM results in their further treatment plan. We try to convince them of the study relevance by sending information material, directing them to our study website, and thorough conversation with the participant. Second, patients without direct or indirect access to an e-mail address cannot be included in the study. We hope to minimize this effect by involving their relatives. Third, there is no empirical evidence on critical PROM values at specific points in time yet. We have tried to overcome this limitation with the formation of an expert consortium and a real data check. Nevertheless, the defined critical values might be over- or underestimated, thus including participants with non-critical recovery paths or missing participants with critical recovery paths. Fourth, participants in the intervention group might have a learning curve when responding to the follow-up questionnaires, deliberately avoiding interventions. From our point of view, this effect is unlikely as the participant does not profit from avoiding interventions. Finally, differences between study centers in personnel, organization, and infrastructure might influence the patient pathway during hospital stay despite adherence to the study protocol.

### Dissemination

The results of this study will be submitted for publication in relevant journals, presented in relevant conferences, and used in the political discourse. Results will play a role in conversations with the sickness funds and the Federal Joint Committee for developing German-wide implementation. Participating hospitals will be included in the publications and have the right for publication of their data. All results will be on an aggregated basis and cannot be connected to individual participants.

## Trial status

The current protocol is version 1, dated January 15, 2019. Recruitment of patients began, dependent on the study center, between October 1 and January 1, 2020 and will end around December 31, 2020. The trial was still open at the time of study protocol submission. The study is expected to run until March 31, 2023.

## Supplementary information

**Additional file 1.** Informed consent.

## Data Availability

Data sets generated and collected by this study will not be publicly available. Analyses of the collected data will be granted only to the researching institute, the evaluating institute, and the participating hospitals.

## Abbreviations

CEA: Cost-effectiveness analysis
EQ-5D-5L: EuroQoL 5-dimension 5-level
HOOS-PS: Hip disability and Osteoarthritis Outcome Score-Physical Function Short-form
KOOS-PS: Knee injury and Osteoarthritis Outcome Score-Physical Function Short-form
PROM: Patient-reported outcome measure
PROMIS: Patient‐Reported Outcomes Measurement Information System
RCT: Randomized controlled trial
THR: Total hip replacement
TKR: Total knee replacement
TU Berlin: Technical University Berlin

## References

[1] Statistical offices of the Federation and the federal states. Diagnosis-Related Groups Statistic 2016, On-Site, Version 0.

[2] Pabinger C, Geissler A (2014). Utilization rates of hip arthroplasty in OECD countries. Osteoarthr Cartil.

[3] Pabinger C, Lothaller H, Geissler A (2015). Utilization rates of knee-arthroplasty in OECD countries. Osteoarthr Cartil.

[4] Pabinger C, Lothaller H, Portner N, Geissler A (2018). Projections of hip arthroplasty in OECD countries up to 2050. Hip Int.

[5] Pross C, Geissler A, Busse R (2017). Measuring, Reporting, and Rewarding Quality of Care in 5 Nations: 5 Policy Levers to Enhance Hospital Quality Accountability. Milbank Q.

[6] Valderas JM, Kotzeva A, Espallargues M, Guyatt G, Ferrans CE, Halyard MY (2008). The impact of measuring patient-reported outcomes in clinical practice: a systematic review of the literature. Qual Life Res.

[7] Homm R, Klauber J, Dormann F (2017). Patient-reported Outcomes (PROs) bei Hüft-und Kniegelenkersatz. Qualitätsmonitor 2017.

[8] Gutacker N, Siciliani L, Moscelli G, Gravelle H (2016). Choice of hospital: Which type of quality matters?. J Health Econ.

[9] Baker PN, Deehan DJ, Lees D, Jameson S, Avery PJ, Gregg PJ (2012). The effect of surgical factors on early patient-reported outcome measures (PROMS) following total knee replacement. J Bone Joint Surg Br.

[10] İmren Y, Dedeoğlu SS, Çakar M, Çabuk H, Bayraktar TO, Gürbüz H (2017). Infrapatellar Fat Pad Excision during Total Knee Arthroplasty Did Not Alter the Patellar Tendon Length: A 5-Year Follow-Up Study. J Knee Surg.

[11] Resolution of the Federal Joint Committee on the Acceptance of the Draft Conceptual Draft of the Institution pursuant to Section 137a of the German Social Code, Book V for a Quality Assurance Procedure for Discharge Management; 2017.

[12] Basch E, Deal AM, Dueck AC, Scher HI, Kris MG, Hudis C (2017). Overall Survival Results of a Trial Assessing Patient-Reported Outcomes for Symptom Monitoring During Routine Cancer Treatment. JAMA.

[13] Basch E, Deal AM, Kris MG, Scher HI, Hudis CA, Sabbatini P (2016). Symptom Monitoring With Patient-Reported Outcomes During Routine Cancer Treatment: A Randomized Controlled Trial. J Clin Oncol.

[14] Busse R, Blümel M, Knieps F, Bärnighausen T (2017). Statutory health insurance in Germany: a health system shaped by 135 years of solidarity, self-governance, and competition. Lancet.

[15] Singh JA, Lewallen DG (2014). Depression in primary TKA and higher medical comorbidities in revision TKA are associated with suboptimal subjective improvement in knee function. BMC Musculoskelet Disord.

[16] Jairath N, Weinstein J (1994). The Delphi methodology (Part one): A useful administrative approach. Can J Nurs Adm.

[17] Choi SW, Schalet B, Cook KF, Cella D (2014). Establishing a common metric for depressive symptoms: linking the BDI-II, CES-D, and PHQ-9 to PROMIS depression. Psychol Assess.

[18] Rudmik L, Drummond M (2013). Health economic evaluation: important principles and methodology. Laryngoscope.

[19] Shwartz M, Restuccia JD, Rosen AK (2015). Composite Measures of Health Care Provider Performance: A Description of Approaches. Milbank Q.

[20] Swart E, Gothe H, Geyer S, Jaunzeme J, Maier B, Grobe TG (2015). Gute Praxis Sekundärdatenanalyse (GPS): Leitlinien und Empfehlungen. Gesundheitswesen.

[21] Quality contracts according to § 110a SGB V - Evaluation concept for investigating the development of quality of care according to § 136b para. 8 SGB V; 2017.

