# Supplementary material for: The use of digitally collected patient-reported outcome measures for newly operated patients with total knee and hip replacements to improve post-treatment recovery: study protocol for a randomized controlled trial
Source: Trials. 2020 Apr 9;21:322. doi: 10.1186/s13063-020-04252-y (PMC7147006; doi:10.1186/s13063-020-04252-y)
Supplement: Supplementary file 1 — Additional file 1. Informed consent. [file 13063_2020_4252_MOESM1_ESM.pdf]

Please store pages 1 and 2 separately

Informed Consent V2 from 18 October 2019

for participation in the scientific study: "PROMoting Quality - Intersectoral Use of Patient Reported Outcome Measures to Increase Patient-Relevant Outcome Quality" of the project partners: Technische Universität (TU) Berlin, aQua Institute for Applied Quality Improvement and Research in Health Care, BARMER, BKK Dachverband eV and HRTBT Medical Solutions GmbH.

PATIENTS – Study Participation

With your consent, you agree to the collection and storage of your survey and medical data and to the processing of said data in pseudonymized form by the aQua Institute and the TU Berlin for the conduction of the study intervention and for research purposes. In addition, you give consent to be contacted by the study assistant via e-mail.

I,

………………………………	………………………………	………………………………,
**First Name** (please print)	**Last Name** (please print)	**Email Address**

hereby declare that Ms. / Mr. ………………………………………………

[First and last name of the study assistant]

informed me, both orally and in writing, about the nature, meaning and risks of the scientific investigations that will be conducted as part of the abovementioned study, and I had sufficient time to clarify questions. I agree to be contacted by the study assistant during my follow-up. Furthermore, I agree to the secure forwarding of my evaluation results by the study assistant to my treating physician following my renewed oral consent.

I understand that I have the right to **withdraw my consent at any time without mentioning a reason and without facing any negative consequences*[1]** and to object to the further processing of my data and demand their destruction.

I voluntarily agree to participate in the 'PROMoting Quality' study.

☐ Yes *[please check box, if applicable]*

I agree

- to my personal data required for the purpose of the abovementioned study being collected, stored in pseudonymized form and processed by the study assistant, including on electronic data carriers, as described in the study information document from *September [version date]*;
- to the publication of my study results in anonymized form, that cannot be traced back to me;
- to the transfer of my pseudonymized study data for the purpose of the abovementioned study to:

Technische Universität Berlin Fachgebiet Management im Gesundheitswesen
Straße des 17. Juni 135. 10623 Berlin Telefon +49 (0)30-314-26933 mig@tu-berlin.de

aQua – Institut für angewandte Qualitätsförderung und Forschung im Gesundheitswesen GmbH
Maschmühlenweg 8-10. 37073 Göttingen Telefon (+49) 0551-789 52- 0 office@aqua-institut.de

………………………………	………………………………	………………………………………
**Place, Date**	**Name** (please print)	**Signature of the Participant**

*[1] Declarations of withdrawal may be given orally to your study assistant or sent in writing to the address below.

PATIENTS – Processing of the Routine Data from the Statutory Health Insurance

In addition to your participation in the study, you have the option to consent to the pseudonymized collection, processing and linking of your personal routine data from the statutory health insurance, provided you are insured with one of the following health insurance funds:

- **BARMER**
- **Betriebskrankenkasse** (to find out which member health insurance funds are participating, please consult the attached list or ask your study assistant)

With your consent, you agree to the use of your personal identification features to identify you as a study participant and to subsequently pseudonymize the routine data from the health insurance fund. Furthermore, you agree to the transfer of your health insurance data, survey data and hospital case data to the aQua Institute, where they will be linked.

I agree to the sending of this page with my health insurance number .... *[enter your health insurance number here]* **to my health insurance fund** .... *[enter the name of your health insurance fund here]*. **Furthermore, I agree to the collection, pseudonymization, transfer, processing and linking of my project-related routine data for research purposes.**

☐ Yes ☐ No

PATIENTS – Processing of the Hospital Case Data

In addition to your participation in the study, you have the option to consent to the pseudonymized collection, processing and linking of your hospital case data. These are data related to your case that are routinely collected during your inpatient stay in the hospital.

With your consent, you agree to your hospital case data being sent in pseudonymized form by the hospital to the aQua Institute for evaluation. You further agree to the linking of your hospital case data to the survey data as well as to selected cost data about you that is stored by your health insurance fund during the study period.

I agree to the collection, pseudonymization, processing and linking of project-related hospital case data for research purposes:

☐ Yes ☐ No

STUDY ASSISTANT

I hereby declare that on .... *[Date of the information session]* I informed the participant, both orally and in writing, of the nature, meaning and risks of the abovementioned study, answered all questions and handed him/her a copy of the study information document and of the informed consent. The medical and legal responsibility remains with the principal investigator.

....	....	....
**Place, Date**	**Name** (please print)	**Signature of the Study Assistant**
